# Candida albicans Hyphal Expansion Causes Phagosomal Membrane Damage and Luminal Alkalinization

**DOI:** 10.1128/mBio.01226-18

**Published:** 2018-09-11

**Authors:** Johannes Westman, Gary Moran, Selene Mogavero, Bernhard Hube, Sergio Grinstein

**Affiliations:** a Program in Cell Biology, The Hospital for Sick Children, Toronto, Canada; b School of Dental Science, Trinity College Dublin, Dublin, Ireland; c Department Microbial Pathogenicity Mechanisms, Hans Knoell Institute, Jena, Germany; d Friedrich-Schiller-University Jena, Jena, Germany; e Department of Biochemistry, University of Toronto, Toronto, Canada; University of Michigan—Ann Arbor

**Keywords:** Candida albicans, candidalysin, *ECE1*, alkalinization, ammonia, dual-wavelength radiometric imaging, hypha, macrophages, pH, phagocytosis, phagosome, yeast-to-hypha transition

## Abstract

Macrophages rely on phagosomal acidity to destroy engulfed microorganisms. To survive this hostile response, opportunistic fungi such as Candida albicans developed strategies to evade the acidic environment. C. albicans is polymorphic and able to convert from yeast to hyphae, and this transition is required to subvert the microbicidal activity of the phagosome. However, the phagosomal lumen, which is acidic and nutrient deprived, is believed to inhibit the yeast-to-hypha transition. To account for this apparent paradox, it was recently proposed that C. albicans produces ammonia that alkalinizes the phagosome, thus facilitating yeast-to-hypha transition. We reexamined the mechanism underlying phagosomal alkalinization by applying dual-wavelength ratiometric pH measurements. The phagosomal membrane was found to be highly permeable to ammonia, which is therefore unlikely to account for the pH elevation. Instead, we find that yeast-to-hypha transition begins within acidic phagosomes and that alkalinization is a consequence of proton leakage induced by excessive membrane distension caused by the expanding hypha.

## Abstract (precis)

C. albicans is the most common cause of nosocomial fungal infection, and over 3 million people acquire life-threatening invasive fungal infections every year. Even if antifungal drugs exist, almost half of these patients will die. Despite this, fungi remain underestimated as pathogens. Our study uses quantitative biophysical approaches to demonstrate that yeast-to-hypha transition occurs within the nutrient-deprived, acidic phagosome and that alkalinization is a consequence, as opposed to the cause, of hyphal growth.

## INTRODUCTION

Candida albicans is a commensal yeast of humans but is frequently the source of mucosal infections and can, in severe cases, cause life-threatening systemic infections (1). It colonizes the epithelial surfaces of 30 to 70% of healthy individuals, and superficial infections are usually transient (2). Due to an aging population, an increased use of antibiotics, and immunocompromising drug treatments, nosocomial C. albicans infections have increased dramatically over the last few decades (3). Unlike many other pathogenic microbes, C. albicans is polymorphic and grows as budding yeast, pseudohyphae, or true filamentous hyphae. The yeast-to-hypha transition is initiated as a response to various environmental stimuli. These include increased pH or temperature, nutrient deprivation, contact with immune cells, and exposure to serum proteins (4, 5). C. albicans yeast cells are associated with commensal growth (but also with dissemination via the bloodstream); by contrast, hyphae are capable of invading epithelia, endothelia, and organ tissues and are thus essential for pathogenicity (4, 5).

C. albicans colonization of the gut is restricted by the bacterial microbiota but also by the immune system, notably patrolling phagocytes. To prevent microbial dissemination from the gut, macrophages and neutrophils quickly recognize and engulf invading microbes through phagocytosis. After engulfment of microbial cells by macrophages, the nascent phagosome undergoes a series of fusion and fission events with endosomes and lysosomes, a phenomenon referred to collectively as “phagosome maturation” (see reference 6 for a review). Fusion of the nascent phagosome with endosomes and lysosomes induces a progressive luminal acidification. This is attributed to the gradual acquisition of vacuolar proton ATPases (V-ATPases) from endosomal compartments. The prevailing phagosomal pH dictates the efficiency of microbial killing and antigen presentation, as well as the degradation of the ingested prey (7).

After phagocytosis of C. albicans yeast, the fungus is confined within the mature phagosome. Nonetheless, at least *in vitro*, C. albicans can escape as a result of intraphagosomal hyphal formation (8–10). It is currently believed that the yeast-to-hypha transition is inhibited within acidic phagosomes, and consequently, C. albicans is thought to manipulate the phagosomal pH prior to hypha formation (11–18). Thus, the ability of C. albicans to alkalinize the phagosome is considered crucial for survival and escape from the macrophage.

Recent studies have proposed that phagosomal alkalinization is a consequence of ammonia (NH_3_) release by C. albicans (15–18). NH_3_ can in principle alkalinize the phagosome by consumption of protons and formation of ammonium (NH_4_^+^). It has been further proposed that, after sufficient NH_3_ production and associated proton consumption, hyphal formation can occur, followed by eventual escape from the phagosome and ultimately from the macrophage itself. Besides C. albicans, NH_3_ generation and protonation have also been suggested to be the cause of phagosome alkalinization for other pathogens such as Mycobacterium tuberculosis and Helicobacter pylori (19–22).

To effectively mediate phagosomal alkalinization, NH_3_ production has to exceed the rate of proton pumping by the V-ATPases. Moreover, and most importantly, the rate of NH_3_ generation has to exceed the rate at which NH_3_ diffuses out of the phagosome. In this regard, it is noteworthy that most mammalian membranes are highly permeable to NH_3_ (23–27). Validation of the alkalinizing role of NH_3_ therefore requires quantitative comparison of these parameters.

A second mechanism that could affect phagosomal pH, which is not mutually exclusive with the generation of NH_3_, is proton exit from the phagosome via candidalysin (28). This pore-forming toxin is a hydrophobic, alpha-helical peptide secreted by the C. albicans hypha after cleavage of the polyprotein Ece1 (28, 29). Candidalysin has been shown to intercalate into membranes and to form pores, leading to lysis of epithelial cells. However, its role during interaction with macrophages and its potential ability to permeabilize the phagosomal membrane have not been investigated.

In this study, we analyzed the role of NH_3_ in phagosome alkalinization by C. albicans. By applying dual-wavelength ratiometric fluorescence imaging, we undertook measurements of phagosomal buffering power, rate of proton pumping, and phagosomal NH_3_ permeability and compared them to the rate of NH_3_ production by C. albicans. Our results suggest that candidalysin does not appear to have a significant role in the formation of hyphae or in fungal escape from the phagolysosome but that hyphal growth itself provoked phagosomal alkalinization by distending the phagosomal membrane.

## RESULTS

### The rate of proton pumping by V-ATPase surpasses the rate of NH_3_ production by C. albicans.

**FIG 1:**
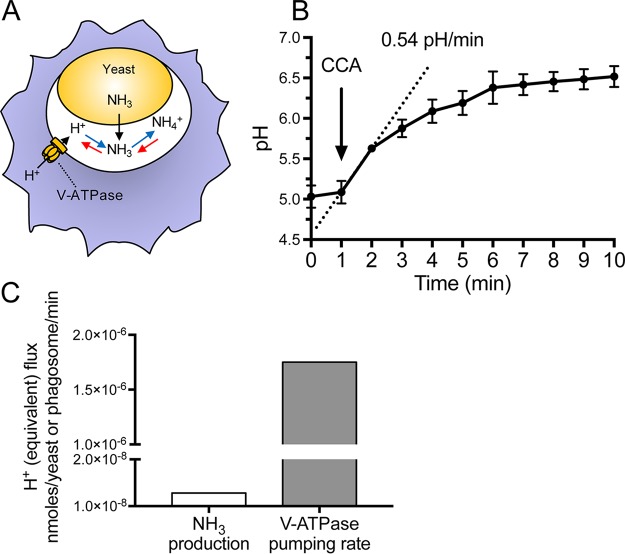
The rate of proton pumping by the V-ATPase exceeds the rate of NH_3_ production. (A) Schematic model illustrating proton pumping by the V-ATPase, NH_3_ production by the yeast, and its protonation to NH_4_^+^. (B) Phagosomal pH measurements acquired by fluorescence ratio imaging. After the baseline pH was recorded, concanamycin A was added where indicated and pH was measured for a further 9 min. The initial rate of ΔpH, estimated from the slope of the dotted line, is shown. Data are means ± SEMs from 16 determinations in 4 independent experiments. (C) Comparison of the rates of NH_3_ production and V-ATPase pumping. Proton pumping was calculated from experiments like that in panel B, as described in Materials and Methods, while the rate of NH_3_ production was derived from reference 15.

For NH_3_ generation by C. albicans to account for macrophage phagosome alkalinization, it would need to exceed the rate of proton pumping by the phagosomal V-ATPases (Fig. 1A). We calculated proton pumping by measuring the rate of change of pH (ΔpH/Δt) induced by addition of the potent and specific inhibitor concanamycin A (CCA) to the murine macrophage cell line RAW 264.7 (here referred to as RAW cells). Such measurements are based on the notion that, in the steady state, the rate of pumping by the V-ATPases is identical to the rate of proton (equivalent) leakage (30). To measure the phagosomal pH, C. albicans yeast cells were allowed to bind fluorescein isothiocyanate (FITC)-labeled concanavalin A and a C. albicans-specific IgG prior to phagocytosis. Such labeled and opsonized yeast cells were centrifuged onto macrophages grown on glass coverslips to initiate phagocytosis synchronously, and at the desired times, phagosomal pH was measured by dual-wavelength ratiometric fluorescence imaging as detailed in Materials and Methods.

As illustrated in Fig. 1B and when added 1 h after phagocytosis—when the phagosomes are acidic, averaging a pH of 5.03 ± 0.14 (mean ± standard error of the mean [SEM] from 32 determinations in 4 experiments)—CCA elicited a rapid alkalinization at an average rate of 0.54 pH/min. The amount of protons pumped per unit time can be calculated by multiplying this rate by the phagosomal buffering capacity. The latter was measured by pulsing the cells with known amounts of membrane-permeant weak electrolytes (see Materials and Methods and reference 31). In 4 independent experiments, the phagosomal buffering power averaged 91.2 ± 3.3 mmol/liter/pH. The rate of proton pumping at the steady state was therefore calculated to be 49.2 ± 15.5 mmol/liter/min.

We proceeded to compare the rate of pumping with the reported rate of NH_3_ production by C. albicans. Vylkova and Lorenz (15) reported a production of ≈35 ppm over 24 hours, which is equivalent to 1.28 × 10^−8^ nmol/yeast/min. Similar rates have been reported by others (13, 14, 16–18). This rate is 2 orders of magnitude lower than the rate of proton pumping at the steady state (Fig. 1C). It should be noted that the activity of the V-ATPase decreases markedly as the pH becomes more acidic (32), so that the disparity between the rates of pumping and NH_3_ production would become even greater at more alkaline pH. At such pH values, the rate of leakage of proton equivalents by other (endogenous) pathways decreases, which would further offset the rates of acidification and alkalinization. We conclude that NH_3_ production by C. albicans is unlikely to account for the reported phagosomal alkalinization.

### C. albicans-containing phagosomes are permeable to NH_3_.

**FIG 2:**
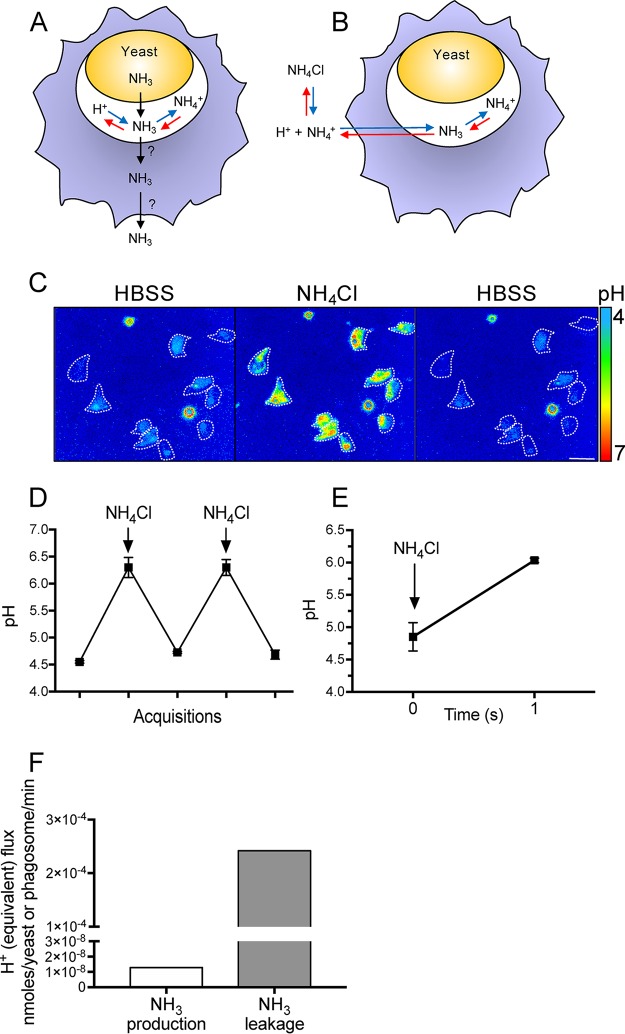
Phagosomes containing C. albicans are highly permeable to NH_3_. (A) Schematic model illustrating C. albicans production and secretion of NH_3_, which can be protonated to form NH_4_^+^. The possibility that NH_3_ diffuses out of the phagosome and the cell is considered. (B) Schematic model illustrating the possible fate of NH_3_ following addition of NH_4_Cl to the extracellular milieu (blue arrows) and its subsequent removal (red arrows). (C) Representative pseudocolor images showing the phagosomal pH before (left), during (middle), and after (right) bathing cells in medium containing 15 mM NH_4_Cl. Bar, 15 μm. (D) Measurement of phagosomal pH during repeated exposure to and removal of extracellular NH_4_Cl. Data are means ± SEMs from 24 determinations in 4 independent experiments. (E) The phagosomal pH was measured before and 1 s after addition of 15 mM NH_4_Cl. Data are means ± SEMs from at least 15 determinations in 4 independent experiments for each type. (F) Comparison of the rates of NH_3_ production and NH_3_ leakage. The latter was calculated from experiments like those illustrated in panel D, as described in Materials and Methods.

Not only is the rate of NH_3_ production insufficient to overcome the rate of proton pumping, but sustained alkalinization would require retention of NH_3_ within the phagosome. Because, as illustrated diagrammatically in Fig. 2A, the protonation of NH_3_ is a rapidly reversible reaction, a fraction of the NH_3_/NH_4_^+^ will always exist inside phagosomes as the unprotonated weak base. Because the extracellular space and the cytoplasm are nominally free of NH_3_/NH_4_^+^, the prevailing outward gradient would promote ongoing loss of NH_3_ from the phagosome, provided that the phagosomal membrane is permeable to the weak base. While most mammalian membranes are permeable to NH_3_, the permeability of the C. albicans-containing phagosome has not been ascertained. We assessed permeability to NH_3_ by measuring the phagosomal pH while pulsing the medium with extracellular NH_3_/NH_4_^+^, as illustrated in Fig. 2B. Addition of NH_3_/NH_4_^+^ to the medium caused an immediate and pronounced phagosomal alkalinization (Fig. 2C to E), ostensibly due to permeation by NH_3_ and protonation to NH_4_^+^. Of note, instantaneous removal of extracellular NH_3_/NH_4_^+^ resulted in rapid restoration of the acidic pH, implying very rapid conversion of NH_4_^+^ to NH_3_ and efflux of the latter (Fig. 2C to E). Such experiments enabled us to estimate the rate at which NH_3_ permeates the membrane of C. albicans-containing phagosomes. Considering the ΔpH elicited by NH_3_/NH_4_^+^ (from 4.85 ± 0.22 to 6.0 ± 0.03, *n *=* *4) (Fig. 2E) after 1 s—the fastest time measurable using our experimental setup—and the buffering power determined earlier, we estimated that NH_3_ can enter/exit phagosomes at a rate of 2.42 × 10^−4^ nmol/phagosome/min. As illustrated graphically in Fig. 2F, this rate is several orders of magnitude greater than the reported rate of NH_3_ production by C. albicans. These additional data reinforce our conclusion that NH_3_ production by C. albicans is unlikely to account for the reported phagosomal alkalinization.

### C. albicans hyphal expansion drives phagosomal alkalinization.

**FIG 3:**
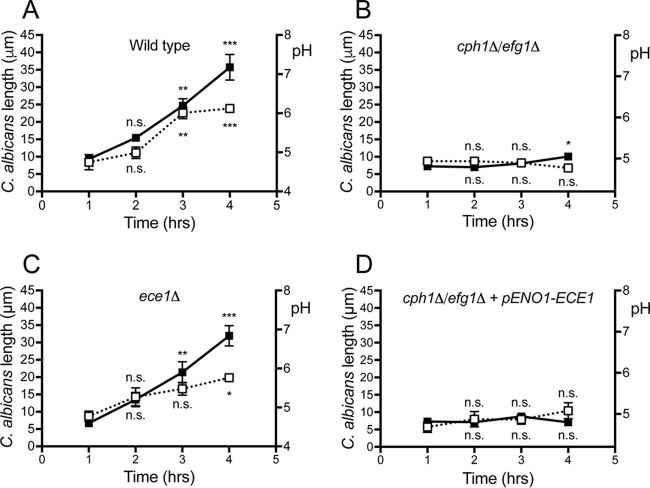
Phagosome alkalinization is associated with hyphal extension. (A to D) Phagosomal pH (right *y* axis, open boxes) and maximal length (left *y* axis, solid black boxes) were measured as a function of time following phagocytosis of the indicated C. albicans strains (see Materials and Methods for details). In each instance, at least 30 phagosomes were quantified per time point and at least 3 independent experiments were performed per condition. Data show means ± SEMs. Significance was calculated by one-way ANOVA, with Tukey’s test. *, *P*  ≤ 0.05; **, *P*  ≤ 0.01; ***, *P*  ≤ 0.001; n.s., not significantly different.

We next analyzed the time course of the pH changes undergone by the C. albicans-containing phagosomes (Fig. 3A). While NH_3_ is presumably produced continuously by the yeast, the phagosome initially becomes acidic and remains so for nearly 2 hours (Fig. 3A, open boxes). These observations not only argue once again against a role for NH_3_ production in the pH changes but suggest that an alternative, time-dependent mechanism is involved. Significant alkalinization was detectable only after ≈3 hours. Notably, marked hyphal growth was clearly apparent at this stage: on average, the hyphae reached 24.7 ± 2 µm in length (Fig. 3A, solid black boxes). We therefore hypothesized that hyphae can form inside acidic phagosomes and that phagosomal alkalinization was a consequence of hyphal growth.

To test this hypothesis, we investigated whether the yeast-locked *cph1*Δ/*efg1*Δ C. albicans mutant (33), which is unable to form hyphae, alters the phagosomal pH as does the wild type. Remarkably, over a period of 4 hours yeast-locked C. albicans cells failed to dissipate the phagosomal acidification (Fig. 3B), despite the fact that this strain generates NH_3_ at rates comparable to the wild-type C. albicans (see Fig. S2A in the supplemental material). These data are consistent with the notion that the pH change is a consequence of hyphal growth.

During hypha formation, the expression of several genes is activated (4, 34). One such gene is *ECE1*, the product of which is processed into the pore-forming toxin candidalysin (28). Because of the association between hyphal growth and phagosomal pH changes, we tested whether candidalysin contributes to the alkalinization. The pores formed by the toxin could conceivably cause leakage of proton (equivalents) through the phagosomal membrane. As illustrated in Fig. 3C, a mutant lacking Ece1 (*ece1*Δ), the precursor required for candidalysin generation, caused phagosomal alkalinization at a rate that was only slightly lower than that induced by the wild type. The difference between the two strains was small but statistically significant (Fig. S2B). Of note, the *ece1*Δ mutant formed hyphae that grew inside the phagosome at rates that were similar to the wild type (Fig. S2C).

These results indicate that, while candidalysin aids in alkalinizing the phagosome, its contribution is comparatively small and other factors are involved. To validate this conclusion, we constructed a strain of yeast-locked C. albicans that expresses *ECE1* at levels comparable to those recorded during hyphal growth of wild-type C. albicans and that produced similar quantities of the candidalysin peptide (Data Set S1 and Fig. S1). Despite the continuous production of candidalysin, this strain had negligible effects on phagosomal pH over a 4-h period (Fig. 3D).

10.1128/mBio.01226-18.6 DATA SET S1 LC-MS/MS analysis and protein database search of candidalysin in Ece1-expressing yeast-locked strain (*cph1Δ/efg1Δ + pENO1-ECE1*). Download Data Set S1, XLSX file, 0.04 MB. Copyright © 2018 Westman et al. 2018 Westman et al. This content is distributed under the terms of the Creative Commons Attribution 4.0 International license.

10.1128/mBio.01226-18.1 FIG S1 Expression of C. albicans *ECE1* quantified by qRT-PCR in the indicated strains cultured under hypha-inducing conditions or while maintaining yeast morphology. Gene expression was normalized to the *ACT1* housekeeping gene. Data show fold change ± standard deviation relative to the yeast morphology of the reference strain SC5314 (one-way ANOVA, Dunnett’s test). *, *P* ≤ 0.05; **, *P* ≤ 0.01; ***, *P* ≤ 0.001. Download FIG S1, TIF file, 0.21 MB. Copyright © 2018 Westman et al. 2018 Westman et al. This content is distributed under the terms of the Creative Commons Attribution 4.0 International license.

The results described above suggest that the phagosomal pH change is a direct consequence of hyphal growth. Remarkably, the opposite has previously been proposed: namely, that alkalinization precedes and is required for hyphal growth (15, 35). The latter concept derives from analyses of the pH dependence of C. albicans hyphal growth, which show higher growth rates at more alkaline pH (35–38). Indeed, it has been shown that neutral pH is required for full virulence of C. albicans, as many effectors are activated by proteolytic cleavage at neutral pH (39). However, while we could readily replicate the faster growth of C. albicans at more alkaline pH values, we also noted that hyphal growth does occur *in vitro* at the pH normally attained by phagosomes, i.e., pH 4.5 to 5.0 (Fig. 1 and 3 and Fig. S2).

10.1128/mBio.01226-18.2 FIG S2 (A) Wild-type and yeast-locked C. albicans were incubated for 24 hours in YPD at 30°C, and NH_3_ production was measured in the supernatant. Data show means ± SEMs (unpaired *t* test). (B) Wild-type and *ece1Δ* C. albicans were phagocytosed by RAW cells and phagosomal pH was measured using dual-wavelength ratiometric fluorescence imaging after 4 hours. Data show means ± SEMs (unpaired *t* test). (C) Length of C. albicans wild-type and *ece1Δ* strains was measured over a period of 4 hours. Data show means ± SEMs (unpaired *t* test). (D) RAW cells were incubated for 15 min with CCA (upper panel) or HBSS (lower panel), washed in PBS, and incubated for 3 hours in RPMI-FBS at 37°C. To determine lysosomal alkalinization, RAW cells were incubated with cresyl violet for 5 min, washed with PBS, and imaged by spinning disk confocal microscopy. Bar, 15 μm. (E) Lysosomes of RAW cells were alkalinized with CCA prior to phagocytosis of wild-type C. albicans. Length of wild-type C. albicans was measured after 3 hours in acidic phagosomes and CCA-treated phagosomes and compared to wild-type C. albicans growing extracellularly. *, *P* ≤ 0.05; **, *P* ≤ 0.01; ***, *P* ≤ 0.001. Download FIG S2, TIF file, 1.14 MB. Copyright © 2018 Westman et al. 2018 Westman et al. This content is distributed under the terms of the Creative Commons Attribution 4.0 International license.

### C. albicans hyphal expansion drives phagosomal membrane rupture.

As the hypha expands over time within the phagosome, increasing mechanical tension must be applied on the phagosomal membrane. This could conceivably alter the permeability of the membrane to proton (equivalents) and potentially even cause its rupture.

**FIG 4:**
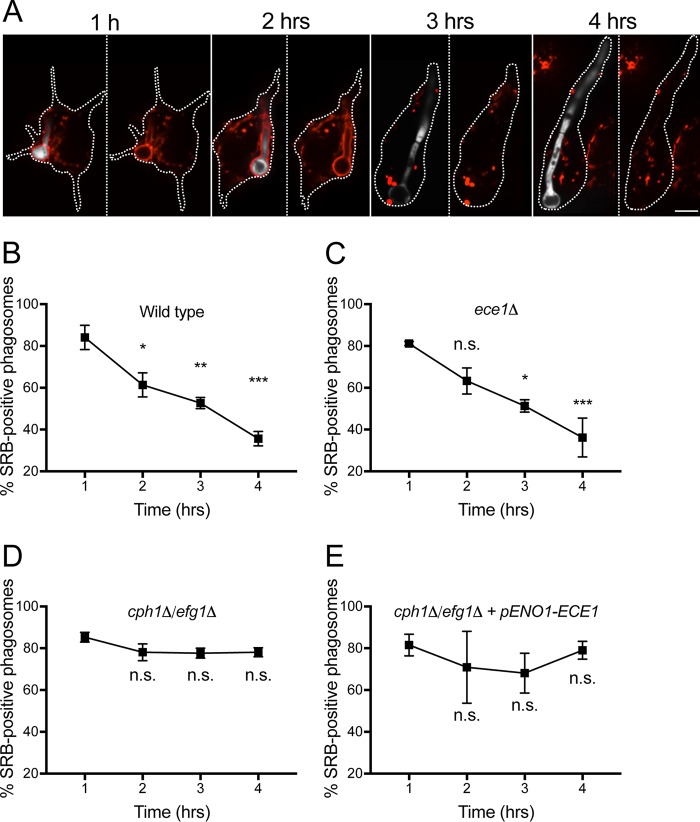
Growth-induced phagosomal leakage demonstrated using sulforhodamine B (SRB). To load endosomes and lysosomes with SRB (red), cells were bathed in 150 μg/ml of the dye 60 min prior to phagocytosis of wild-type C. albicans yeast. (A) During phagosome maturation, endosomes and lysosomes fuse with the phagosome, delivering SRB to its lumen. Cells were imaged over 4 hours following phagocytosis by spinning disk confocal microscopy, and representative images are depicted. C. albicans is shown in white (left images for each time); the yeasts were omitted from the right panels to more clearly visualize the SRB outline. The borders of the macrophages are outlined using a dotted white line. Bar, 5 μm. (B to E) Quantitation of the fraction of phagosomes retaining SRB as a function of time after phagocytosis. Results obtained using wild-type C. albicans (B) or Ece1-null (*ece1*Δ) (C), yeast-locked (*cph1Δ/efg1Δ*) (D), or Ece1-expressing yeast-locked (*cph1Δ/efg1Δ + pENO1-ECE1*) (E) strains are illustrated. Data are means ± SEMs from at least 100 determinations in 3 independent experiments for each type. Significance was calculated using one-way ANOVA, with Tukey’s test. *, *P*  ≤ 0.05; **, *P*  ≤ 0.01; ***, *P*  ≤ 0.001; n.s., not significantly different.

We assessed the phagosomal membrane integrity using sulforhodamine B (SRB), a fluorescent dye that is nominally impermeant across biological membranes. SRB was delivered to phagosomes via fusion with lysosomes, which had been previously loaded with the dye using a pulse-chase protocol (see Materials and Methods for details). As illustrated in Fig. 4A and Movie S1, SRB contained within phagolysosomes can be initially observed lining the yeast and hyphae. Over time, however, the SRB contained within phagosomes formed by wild-type C. albicans was lost progressively (Fig. 4A and B). In line with phagosomal alkalinization, the number of SRB-positive phagosomes decreased over a period of 4 hours (Fig. 4B). SRB was lost at a similar rate from the *ece1*Δ mutant, which grows at a comparable rate as the wild-type C. albicans strain (Fig. 4C). Importantly, SRB was retained during the same period by the yeast-locked mutants, even when they were engineered to produce Ece1 (Fig. 4D and E). The latter data suggest that SRB leakage is a consequence of hyphal growth and not permeation of the dye via candidalysin. Our data are most consistent with a growth-induced change in permeability, likely manifested as rupture of the membrane. Note that, as documented below, in some instances rupture may have been transient, followed by membrane repair.

10.1128/mBio.01226-18.7 MOVIE S1 Growth-induced phagosomal leakage demonstrated using sulforhodamine B (SRB). Download Movie S1, MOV file, 0.04 MB. Copyright © 2018 Westman et al. 2018 Westman et al. This content is distributed under the terms of the Creative Commons Attribution 4.0 International license.

### Phagosomal expansion using GPN causes alkalinization and membrane rupture similar to hyphal expansion.

**FIG 5:**
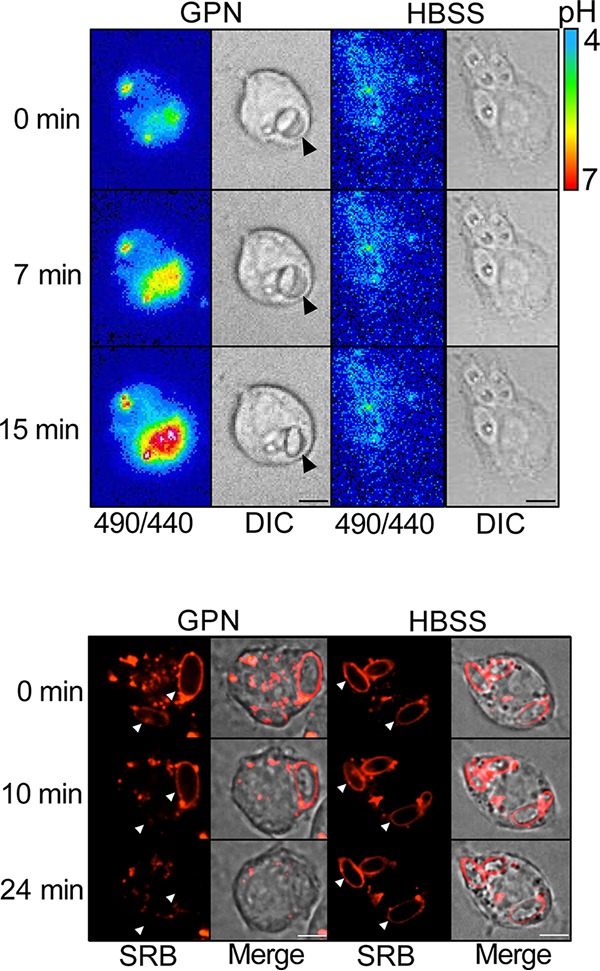
Gly-Phe-β-naphthylamide (GPN) induces expansion-dependent phagosomal alkalinization and membrane rupture. (Top) One hour after phagocytosis of yeast-locked C. albicans, phagosomal expansion was induced by adding 100 μM GPN. Phagosomal pH was recorded every 15 s by fluorescence imaging, measuring the ratio of the emission obtained when exciting at 490 nm versus 440 nm. Vehicle alone (HBSS) was used as negative control. Bar, 5 μm. (Bottom) Endosomes and lysosomes were loaded with SRB (red) by incubating cells with 150 μg/ml of the dye for 60 min prior to phagocytosis. One hour after phagocytosis of yeast-locked C. albicans, phagosomal expansion was induced by adding 200 μM GPN (left panel). HBSS was used as negative control. DIC and fluorescence images were acquired every minute for 25 min. Arrowheads point to phagosomes. Bar, 5 μm.

To verify that mechanical tension (like that induced by hyphal growth) suffices to produce phagosomal alkalinization and membrane rupture, we treated phagosomes containing the yeast-locked C. albicans *cph1*Δ/*efg1*Δ mutant with Gly-Phe-β-naphthylamide (GPN). GPN is a membrane-permeant dipeptide and a substrate for cathepsin C. Phagosomal cathepsin C can cleave GPN, generating the membrane-impermeant Phe-β-naphthylamide. Accumulation of Phe-β-naphthylamide drives osmotically obliged water into the phagosome, which consequently expands, exerting hydrostatic pressure that distends the membrane in a manner akin to that occurring during hyphal growth. As illustrated in Fig. 5 (top, left panel) and Movie S2, following addition of GPN, phagosomes containing the yeast-locked C. albicans expanded rapidly and alkalinized within 20 min, while untreated phagosomes remained acidic (Fig. 5, top, right panel, and Movie S2). Even faster onset of alkalinization was observed using higher concentrations of GPN (data not shown). GPN also induced leakage of phagosomal SRB within minutes (Fig. 5, bottom, left panel, and Movie S3), while untreated phagosomes remained SRB positive (Fig. 5, bottom, right panel, and Movie S3). We concluded that mechanical tension is sufficient to cause phagosome alkalinization and membrane rupture.

10.1128/mBio.01226-18.8 MOVIE S2 Gly-Phe-β-naphthylamide (GPN) induces expansion-dependent phagosomal alkalinization after phagocytosis of yeast-locked C. albicans. Nontreated phagosomes are used as a control. Download Movie S2, AVI file, 0.04 MB. Copyright © 2018 Westman et al. 2018 Westman et al. This content is distributed under the terms of the Creative Commons Attribution 4.0 International license.

10.1128/mBio.01226-18.9 MOVIE S3 Gly-Phe-β-naphthylamide (GPN) induces expansion-dependent phagosomal membrane rupture after phagocytosis of yeast-locked C. albicans. Nontreated phagosomes are used as a control. Download Movie S3, AVI file, 0.04 MB. Copyright © 2018 Westman et al. 2018 Westman et al. This content is distributed under the terms of the Creative Commons Attribution 4.0 International license.

### Phagosomes undergo transient changes in pH before irreversible rupture.

**FIG 6:**
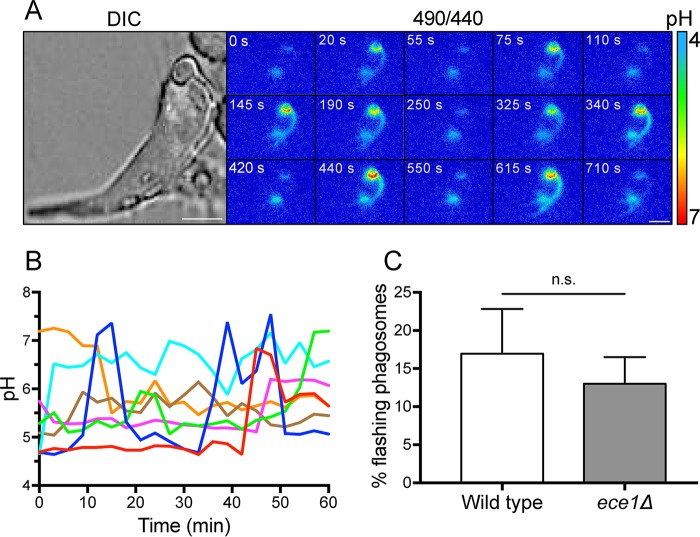
C. albicans phagosomes display transient proton leakage prior to undergoing irreversible breaks. (A) Phagosomal pH was measured 3 hours postinfection by dual-wavelength ratio imaging. Images were acquired every 5 s for 15 min. An enlarged DIC image of the macrophage analyzed is shown in the left panel. Bars, 5 μm. (B) Different patterns of transient proton leakage recorded over 60 min. Each individually colored trace represents a separate phagosome. (C) Quantitation of the fraction of phagosomes recorded in each field of view that displayed flashing events during a 10-min period of analysis, 3 hours postinfection. Data show means ± SEMs from at least 30 determinations in 3 independent experiments of each type. Statistical significance was assessed by an unpaired *t* test. n.s., not significantly different.

Biological membranes have effective means of repairing occasional tears, thereby maintaining homeostasis (40–44). It was therefore conceivable that phagosomes containing growing hyphae would attempt to maintain their integrity, at least until the repair mechanisms were exhausted. To investigate this possibility, macrophages were infected with C. albicans, and after hyphae were allowed to grow for 3 hours, we repeatedly measured phagosomal pH every 5 s over a period of 10 to 60 min. As depicted in Fig. 6A, acidic phagosomes frequently underwent a series of rapid changes in pH. We refer to this phenomenon as a pH flash. While phagosomes often underwent one pH flash during the observation period, some phagosomes flashed up to 6 times (Movie S4). The variability of the flashing pattern is illustrated in Fig. 6B, where the time courses of pH recordings from multiple phagosomes are overlaid. The rate of pH flashes was similar when the C. albicans *ece1*Δ mutant was phagocytosed, suggesting that pH flashes are a consequence of hyphal expansion rather than candidalysin (Fig. 6C and Movie S4). The flashing pattern is consistent with the notion that, during hyphal expansion, phagosomes undergo tears that can in some instances be repaired, before irreversible rupture occurs, leading to sustained alkalinization and loss of SRB. It is noteworthy that neither the transient nor the permanent phagosomal breaks were associated with macrophage lysis, as the macrophages remained largely impermeable to propidium iodide (PI) 4 hours postinfection (Fig. S3) (total cell lysis was less than 10%).

10.1128/mBio.01226-18.10 MOVIE S4 Phagosomes containing wild-type or *ece1Δ* C. albicans phagosomes display transient proton leakage (pH flashes). Download Movie S4, AVI file, 0.04 MB. Copyright © 2018 Westman et al. 2018 Westman et al. This content is distributed under the terms of the Creative Commons Attribution 4.0 International license.

10.1128/mBio.01226-18.3 FIG S3 (A) RAW cell viability was measured 4 hours after infection with wild-type C. albicans (blue). Concanavalin A-FITC (green) and propidium iodide (PI; red) were added after 4 hours to label escaping hyphae and dying RAW cells, respectively. Bar, 20 μm. (B) PI-positive RAW cells were quantified at 4 hours after phagocytosis of wild-type C. albicans. Pneumolysin was used as a positive control. Data show means ± SEMs (one-way ANOVA, Tukey’s test). Download FIG S3, TIF file, 2.18 MB. Copyright © 2018 Westman et al. 2018 Westman et al. This content is distributed under the terms of the Creative Commons Attribution 4.0 International license.

## DISCUSSION

In this study, we reached three main conclusions: first, that NH_3_ generation by C. albicans and its retention by phagosomes cannot be responsible for the observed alkalinization; second, that initiation of hyphal growth occurs in acidic phagosomes; and third, that hyphal growth drives phagosomal alkalinization by stretching and eventually rupturing the phagosomal membrane. These conclusions are discussed in turn below.

It is generally agreed that the mature phagosome containing C. albicans is highly acidic, and low pH is known to inhibit yeast-to-hypha transition. Hence, it was recently suggested that C. albicans produces NH_3_ to alkalinize the phagosome prior to hyphal growth (13, 15–18). Our findings suggest that intracellular NH_3_ production by C. albicans is not directly responsible for the alkalinization. The rate of NH_3_ generation is much too low to overcome the ability of the V-ATPase to acidify the phagosome (Fig. 1), and more importantly, NH_3_ cannot be retained within the phagosomes, which are extremely permeable to the uncharged weak base (Fig. 2).

Our findings indicate that, rather than being the cause of hyphal growth, phagosomal alkalinization is its consequence. How then is hyphal extension initiated? Several environmental factors influence yeast-to-hypha transition, including high temperature (37°C), adherence to host cells, high CO_2_, and nutrient deprivation (4, 5). All these factors are experienced by C. albicans cells within the mammalian phagosome and may suffice to induce hyphal formation despite the acidic phagosomal pH, albeit at a reduced rate.

Among the factors listed above, CO_2_ deserves special mention. CO_2_ is known to contribute to the yeast-to-hypha transition as part of the process involving conversion of arginine to urea that is secondarily decomposed to NH_3_ and CO_2_ (9, 45). Indeed, after phagocytosis by macrophages C. albicans changes its transcriptional program, stimulating biosynthesis of l-arginine, the l-arginase Car1p, and several related arginase genes (34) to generate urea. In turn, urea is converted to NH_3_ and CO_2_ by the amidolyases Dur1,2p. Accordingly, l-arginine and urea induced hyphal formation in a C. albicans wild-type strain but not in a *dur1,2Δ* (amidolyase-deficient) strain (9). The *dur1,2Δ* strain also failed to escape from mouse macrophages and was less virulent after intravenous injections in mice (46). While this has been interpreted as supporting a requirement for NH_3_ formation, it may instead reflect a role for CO_2_ or its conjugated weak base, bicarbonate. Bicarbonate, which serves a signaling role in other systems, would accumulate in the fungal cytoplasm supported by the active alkalinization promoted by the plasma membrane H^+^-ATPase (Pma1) (47). Accumulation of bicarbonate would be further aided by alkalinization of the phagosomal lumen. Accordingly, we find that C. albicans hyphae grow faster in phagosomes treated with CCA, a V-ATPase inhibitor that dissipates the lysosomal and phagosomal acidification, which was verified using the acidotrophic dye cresyl violet (see Fig. S2D and E in the supplemental material). Thus, while capable of growing inside acidic phagosomes (Fig. 3), C. albicans hyphae indeed extend more rapidly at more alkaline pH (Fig. S2E).

Some of the evidence supporting the involvement of NH_3_ stemmed from experiments using mutants with defective amino acid permeases, which ostensibly lacked the substrates to generate sufficient NH_3_. One such mutant, the *stp2Δ* strain, was found to be unable to alkalinize phagosomes (15). In the presence of amino acids, Stp2 induces the transcription of genes leading to amino acid uptake and catabolism. This in turn produces urea, which subsequently leads to the production of NH_3_ and CO_2_ by urea amidolyases. It is noteworthy, however, that Stp2 affects the expression of several genes (48, 49) and, as a result, suffers from growth defects, particularly in environments where nutrient availability is restricted, such as the phagosomal lumen. Thus, it is impossible to distinguish whether the effects caused by deletion of the gene are due to lack of NH_3_ production or to impaired growth. Other mutants such as the *ahr1*Δ strain used to buttress the NH_3_ hypothesis suffer from similar shortcomings, which is why we opted not to include them in our analyses (14, 17, 18). Since NH_3_ generation appeared unlikely to account for the observed alkalinization, we sought for an alternative mechanism. Our data are consistent with the notion that hyphal growth distends the phagosomal membrane, causing leakage of proton equivalents and even larger molecules like SRB. In the early stages, the ruptures are transient, possibly reflecting the activation of repair mechanisms; indeed, we have preliminary evidence that reacidification is associated with additional fusion of lysosome-associated membrane protein (LAMP)-positive compartments with the phagosome (data not shown). The progressive membrane tears become irreversible thereafter, judged by the impossibility of reestablishing the acidic pH. Of note, the sudden and initially reversible increases in pH cannot be readily explained by NH_3_ production, which is anticipated to be continuous, producing a gradual and sustained pH change. That mechanical stretching of the phagosomal membrane is the cause of the permeability change is supported by the observation that outward hydrostatic pressure established by osmotic means—using GPN—resulted in a similar disruption of the phagosomal membrane, with dissipation of the pH gradient and leakage of SRB (Fig. 5). Whether transient or more permanent, phagosomal membrane rupture exposes the fungus and its products to the cytosolic milieu. Little is known regarding the means whereby C. albicans-secreted effectors activate signaling pathways within the cytosol. We therefore speculate that discontinuities in the phagosomal membrane associated with hyphal growth could contribute to inflammasome activation and pyroptosis (50–52).

In conclusion, we propose that hyphal growth is initiated inside acidic phagosomes (albeit at a reduced rate) and that alkalinization results from excessive membrane distension, which either activates mechanosensitive channels and/or causes outright rupture of the phagosomal lining. The discontinuities may initially be transient, as the membrane is repaired by fusion with other organelles (likely LAMP-positive late endosomes-lysosomes), but eventually become permanent, leading to sustained alkalinization and granting the fungus access to the richer cytosolic environment.

## MATERIALS AND METHODS

### Strains and reagents.

Experiments were carried out using mouse RAW 264.7 macrophages (ATCC). RAW cells were plated sparsely in 12-well tissue culture plates (Corning Inc.) and grown overnight at 37°C in an air-CO_2_ (19:1) environment in RPMI 1640 (Wisent Inc.) supplemented with 5% (vol/vol) fetal bovine serum (FBS). The C. albicans wild-type strain was the prototrophic strain BWP17/CIp30 (53). Other strains used are listed in Table S2 in the supplemental material. C. albicans cultures were grown in YPD medium (1% yeast extract, 2% peptone, 2% dextrose) at 30°C overnight. Cultures were washed in sterile phosphate-buffered saline (PBS) and adjusted to the required cell density. Concanavalin A labeled with FITC and sulforhodamine B were from Invitrogen. Nigericin, monensin, and Gly-Phe-β-naphthylamide were from Sigma. Concanamycin A was from Abcam. Recombinant pneumolysin was a kind gift from John Brumell.

### Phagocytosis of C. albicans.

After overnight incubation at 30°C, C. albicans yeast was washed twice in PBS and incubated in the dark with concanavalin A-FITC (1:100) and rabbit anti-C. albicans IgG (1:167, for 60 min at room temperature with rotation). After labeling, yeast was washed twice in PBS and diluted to an optical density at 600 nm (OD_600_) of 1.0 in PBS.

Five microliters of concanavalin A-FITC-labeled yeast in fresh RPMI-FBS was added to RAW cells grown on glass coverslips, which were then centrifuged at 1,500 ×* g* for 1 min at room temperature to synchronize phagocytosis. After 20 min of incubation at 37°C, yeast not associated with the macrophages was washed away with PBS and yeast that had adhered to the macrophages but had not been internalized was labeled with donkey anti-rabbit Cy3 (1:1,000) for 10 min at 37°C. Cells were imaged live or, where indicated, fixed with 4% paraformaldehyde for subsequent analysis. All phagocytosis experiments were imaged in Hanks’ balanced salt solution (HBSS).

### Buffering capacity (β).

To determine buffering capacity (β) of the C. albicans-containing phagosome, phagosomes were allowed to acidify for 1 h before phagosomal pH was measured ratiometrically, as described below. The bathing solution was switched to HBSS containing 15 mM NH_4_Cl, and the pH was measured again immediately. At the end of each experiment, a standard calibration was performed as described below and fluorescence ratios were converted to pH. The intraphagosomal NH_4_^+^ concentration ([NH_4_^+^]) was calculated using the Henderson-Hasselbalch equation, and the intrinsic buffer capacity (in millimoles/liter/pH) was calculated as Δ[NH_4_^+^]/ΔpH.

### V-ATPase pumping rate.

Phagosomes were generated as described above, and after 1 h, the steady-state phagosomal pH was measured prior to addition of 2 μM CCA to the bathing solution. Thereafter, the phagosomal pH was measured every minute for 15 min at 37°C. At the end of each experiment, a standard calibration was performed and fluorescence values were converted to pH. The rate of change of the luminal pH (ΔpH) measured during the first minute after CCA treatment was used to estimate the vacuolar H^+^-ATPase (V-ATPase) pumping rate, which was assumed to be identical to the proton leakage rate at steady state. Proton pumping rates were calculated as (ΔpH × β × phagosome volume)/time. To quantify the phagosomal volume, the radius of phagosomes containing C. albicans yeast was measured microscopically. Volume was calculated assuming that the phagosomes were spherical (volume = 4/3 π *r*^3^). Phagosomal and lysosomal alkalinization was confirmed using the acidotrophic dye cresyl violet as previously described (54).

### Determination of NH_3_ leakage rate.

Phagosomes were generated as described above, and after 1 h, the steady-state phagosomal pH was measured prior to the addition of 15 mM NH_4_Cl. Phagosomal pH was then measured every second for 10 s. A standard calibration was generated as described below, to determine the change in pH (ΔpH) induced by NH_3_ addition or withdrawal. Leakage of NH_3_ was calculated as (ΔpH × β × phagosome volume)/time.

### Dual-wavelength ratiometric fluorescence measurements.

RAW cells were allowed to ingest C. albicans as described above, and the coverslips were then mounted in a Chamlide magnetic chamber and overlaid with HBSS. The chamber was placed in a Leiden microincubator maintained at 37°C on the stage of an inverted microscope (DM IRB; Leica Biosystems) equipped with a 40A/1.25-numerical-aperture (NA) oil objective (Leica Biosystems), a lamp (X-Cite 120; EXFO Life Sciences Group), and filter wheels (Sutter Instrument) that control excitation and emission filters. For experiments using concanavalin A-FITC-labeled C. albicans, excitation wavelengths were alternated between 485 ± 10 nm and 438 ± 12 nm, with emitted light selected through a 520-nm filter. Light was captured by a cooled electron-multiplied charge-coupled device camera (Cascade II; Photometrics). The filter wheel and camera were under the control of MetaFluor software (Molecular Devices). Emission at 520 nm from light excited at the two excitation wavelengths was collected, and their ratio was calculated online from phagosomes at different time points. For phagosomal pH measurements, the 490/440-nm ratio of at least 30 phagosomes was measured for each strain and time point. For transient pH oscillations (pH flashes), the 490/440-nm ratio was acquired from the same field of view every 2 s for 10 min or every 3 min for 60 min. For GPN-induced phagosomal expansion, 100 μM GPN was added to phagosomes containing yeast-locked C. albicans 1 h postinfection. GPN-treated phagosomes were visualized for 30 min at 37°C.

### Conversion of dual-wavelength fluorescence ratio to pH.

To convert measured phagosomal fluorescence ratios to pH, samples were sequentially bathed in isotonic K^+^ solutions containing 10 μM nigericin and 5 μM monensin and calibrated to pH 7.5, 6.0, 5.5, 5.0, and 4.5, respectively. Samples were imaged 5 min after addition of each solution to ensure pH equilibration across all compartments. After background subtraction at each wavelength, measured fluorescence ratios at defined pH were plotted into a calibration curve that was fitted with least squares. The measured phagosome fluorescence ratios were transformed into intracellular pH by using the equation describing the curve generated above. For pH measurements, fluorescence values from each phagosome were obtained using the freehand tool in Fiji (version 1.0) to select regions of interest (ROIs) delimiting the phagosome. The fluorescence intensity was corrected by subtracting the background fluorescence at each wavelength and converted to pH by using the equation described above. For pseudocolor display, a RatioPlus plug-in in Fiji was used to depict the 490/440-nm ratio.

### C. albicans hyphal growth.

The length of C. albicans was measured from differential inference contrast (DIC) images acquired at different time points after phagocytosis. Total length included the yeast head and hyphal projection.

### Sulforhodamine B loading and leakage during phagocytosis.

To load endosomes and lysosomes with SRB, RAW cells were bathed for 60 min at 37°C in RPMI-FBS containing 150 μg SRB/ml, prior to phagocytosis. After loading, phagocytosis was initiated as described above. Leakage of SRB from phagosomes was measured using spinning disk confocal microscopy, and the percentage of SRB-positive phagosomes was calculated as (SRB-positive phagosomes/total phagosomes) × 100. For GPN-induced membrane rupture, 200 μM GPN was added to phagosomes containing yeast-locked C. albicans 1 h postinfection. GPN-treated phagosomes were visualized for 30 min by DIC and fluorescence microscopy.

### Spinning disk confocal microscopy.

Confocal images were acquired using a spinning disk system (WaveFX; Quorum Technologies Inc.). The instrument consists of a microscope (Axiovert 200M; ZEI SS), scanning unit (CSU10; Yokogawa Electric Corporation), electron-multiplied charge-coupled device (C9100-13; Hamamatsu Photonics), five-line (405-, 443-, 491-, 561-, and 655-nm) laser module (Spectral Applied Research), and filter wheel (MAC5000; Ludl) and is operated by Volocity software version 4.3.2 or 6.2.1 (Perkin-Elmer). Images were acquired using a 63×/1.4-NA oil objective (Zeiss) coupled to an additional 1.5× magnifying lens and the appropriate emission filter. Cells were maintained at 37°C using an environmental chamber (Live Cell Instruments).

### Construction of an *ECE1*-expressing yeast-locked strain (*cph1Δ/efg1Δ + pENO1-ECE1*) of C. albicans.

The *cph1*Δ/*efg1*Δ + *pENO1*-*ECE1* strain expressing *ECE1* under the control of the C. albicans enolase (*ENO1*) promoter was generated from the parent strain HLC54 (*cph1*Δ/*efg1*Δ) (33). A cassette containing the C. albicans *NAT1* (*CaNAT1*) marker and the *ENO1* promoter was amplified from pNAT-ENO1 (50) using the primers ECE1_ENO1_PF and ECE1_ENO1_PR containing homology to the *ECE1* gene, allowing targeted integration to replace the native *ECE1* promoter (Table S1). Correct integration was confirmed by PCR with the *ENO1*-specific primer ENO1-S in conjunction with a reverse primer internal to the *ECE1* gene (ECE1-IR) (Table S1). Positive transformants yielded a specific product approximately 450 bp in size (data not shown).

10.1128/mBio.01226-18.4 TABLE S1 PCR primers used to generate and verify the Ece1-expressing yeast-locked strain (*cph1Δ/efg1Δ + pENO1-ECE1*). Download Table S1, DOCX file, 0.06 MB. Copyright © 2018 Westman et al. 2018 Westman et al. This content is distributed under the terms of the Creative Commons Attribution 4.0 International license.

10.1128/mBio.01226-18.5 TABLE S2 C. albicans strains used in this study. Download Table S2, DOCX file, 0.10 MB. Copyright © 2018 Westman et al. 2018 Westman et al. This content is distributed under the terms of the Creative Commons Attribution 4.0 International license.

### Quantification of *ECE1* expression by reverse transcriptase-quantitative PCR (qRT-PCR).

Total RNA was extracted from yeast-phase cells grown in YPD medium at 30°C or hyphae grown in RPMI 1640 according to the method described in reference 51. RNA (500 ng) was treated with DNase (Epicentre), and cDNA was synthesized using Superscript III reverse transcriptase (Invitrogen). cDNA samples were used for quantitative PCR with EvaGreen mix (Bio&SELL). Primers (ACT1-F/R and ECE1-F/R, Table S1) were used at a final concentration of 500 nM. qPCR amplifications were performed using a CFX96 thermocycler (Bio-Rad). *ECE1* expression was calculated using the threshold cycle (ΔΔ*C_T_*) method, with *ACT1* as the reference gene and C. albicans reference strain SC5314 (yeast morphology) as the control sample.

Results of three biological replicates were analyzed in Prism version 6 (GraphPad, San Diego, CA).

### LC-MS/MS analysis of hypha-secreted Ece1 peptides.

Analysis of hypha-secreted Ece1 peptides was optimized for the detection of candidalysin and performed as previously described (28). Briefly, *Candida* strains were cultured for 18 h under strong hypha-inducing conditions [YNB medium containing 2% sucrose, 75 mM 3-(*N*-morpholino)-2-hydroxypropanesulfonic acid (MOPSO) buffer, pH 7.2, 5 mM *N*-acetyl-d-glucosamine, 37°C] or non-hypha-inducing conditions (YNB medium containing 2% sucrose, 0.1 M citric acid, 0.1 M trisodium citrate, pH 4, 30°C). Peptides secreted into the exhausted culture medium were enriched by solid-phase extraction (SPE), passed through a 10-kDa-molecular-weight-cutoff filter, and resolubilized in 0.2% formic acid in 71:27:2 (vol/vol/vol) acetonitrile (ACN)-H_2_O-dimethyl sulfoxide (DMSO). Liquid chromatography-tandem mass spectrometry (LC-MS/MS) analysis was performed using an Ultimate 3000 nano-LC coupled to a Q Exactive Plus (Thermo). Peptides were separated on an Accucore C_4_ column (15 cm by 75 µm, 2.6 µm) with a 60-min LC gradient of 0.2% HCOOH in 95:5 H_2_O-DMSO (A) and 0.2% HCOOH in 85:10:5 ACN-H_2_O-DMSO (B) for 0 to 1.5 min at 60% B, 35 to 45 min at 96% B, and 45.1 to 60 min at 60% B. The Top10 precursor ions (full scan at *m/z* 300 to 1,600, *R* = 70,000 full width at half maximum [FWHM]) per scan cycle underwent higher-energy collision dissociation (HCD) fragmentation (30 V). Resulting MS/MS spectra were monitored at *R* = 17.5k (FWHM). Proteome Discoverer 1.4 (Thermo) and the Sequest HT algorithm were used for protein database searching against C. albicans SC5314 (Candida Genome Database [http://www.candidagenome.org]). Mass spectra were searched for both unspecific cleavages (no enzyme) and tryptic peptides up to 4 missed cleavages. The precursor mass tolerance was 10 ppm, and the fragment mass tolerance was 0.02 Da. At least two unique peptides per protein, a false-discovery rate of <1%, and *X*_corr_ validation (from 2.0 at *z* = 2 up to 3.0 at *z* = 6) were required for positive protein hits.

### Statistical analysis.

Unless otherwise indicated, data are presented as means ± SEMs from the number of determinations shown in parentheses. Statistical significance was determined using unpaired *t* test, one-way analysis of variance (ANOVA) (Tukey’s test or Dunnett’s test), and two-way ANOVA (multiple comparisons) with Prism 7 (GraphPad Software), with *P*  < 0.05 considered significant.
