# Supplementary figures and images for: Candida albicans Hyphal Expansion Causes Phagosomal Membrane Damage and Luminal Alkalinization
Source: mBio. 2018 Sep 11;9(5):e01226-18. doi: 10.1128/mBio.01226-18 (PMC6134096; doi:10.1128/mBio.01226-18)

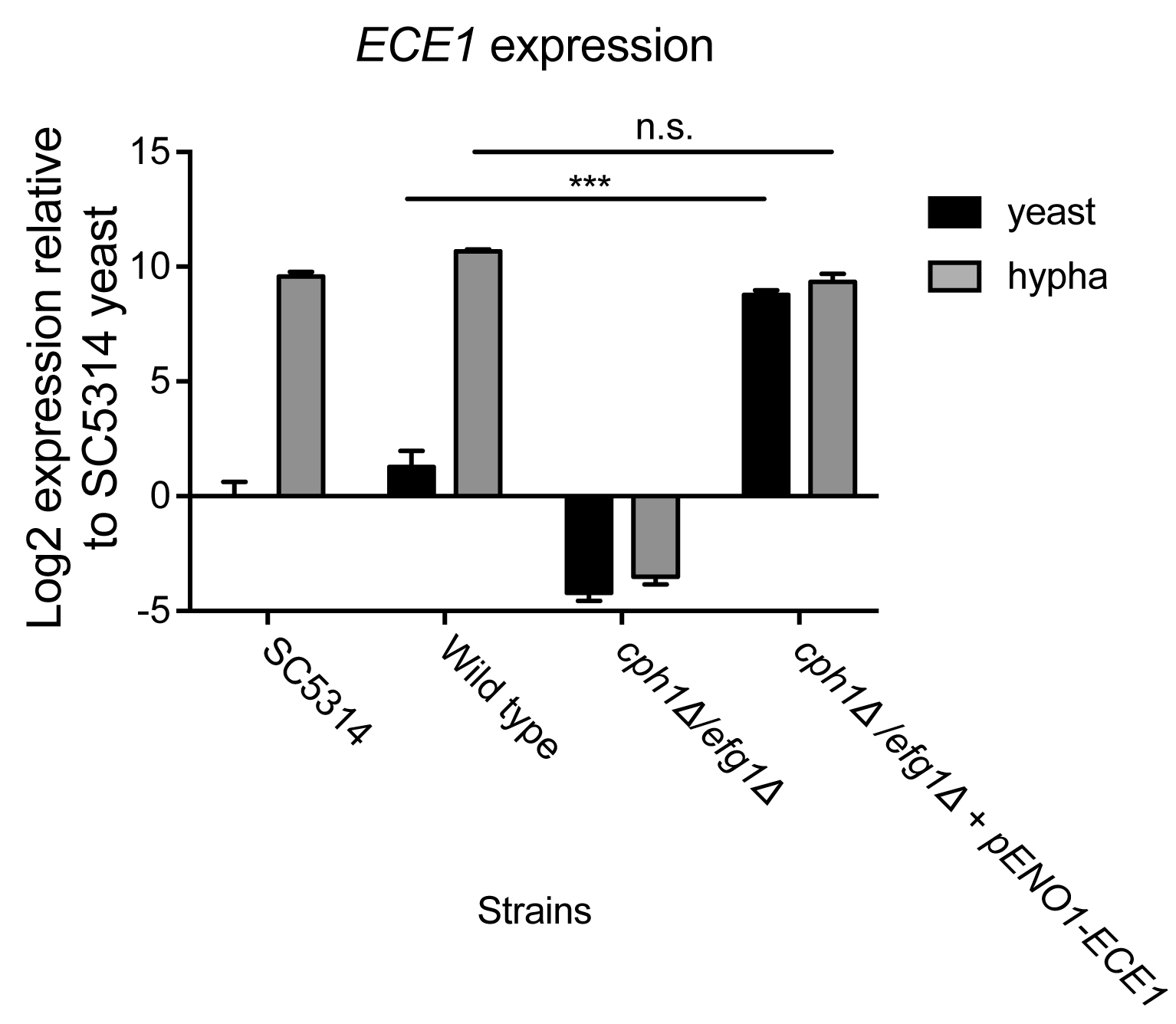

Supplement: FIG S1 [file mbo004184059sf1.tif]

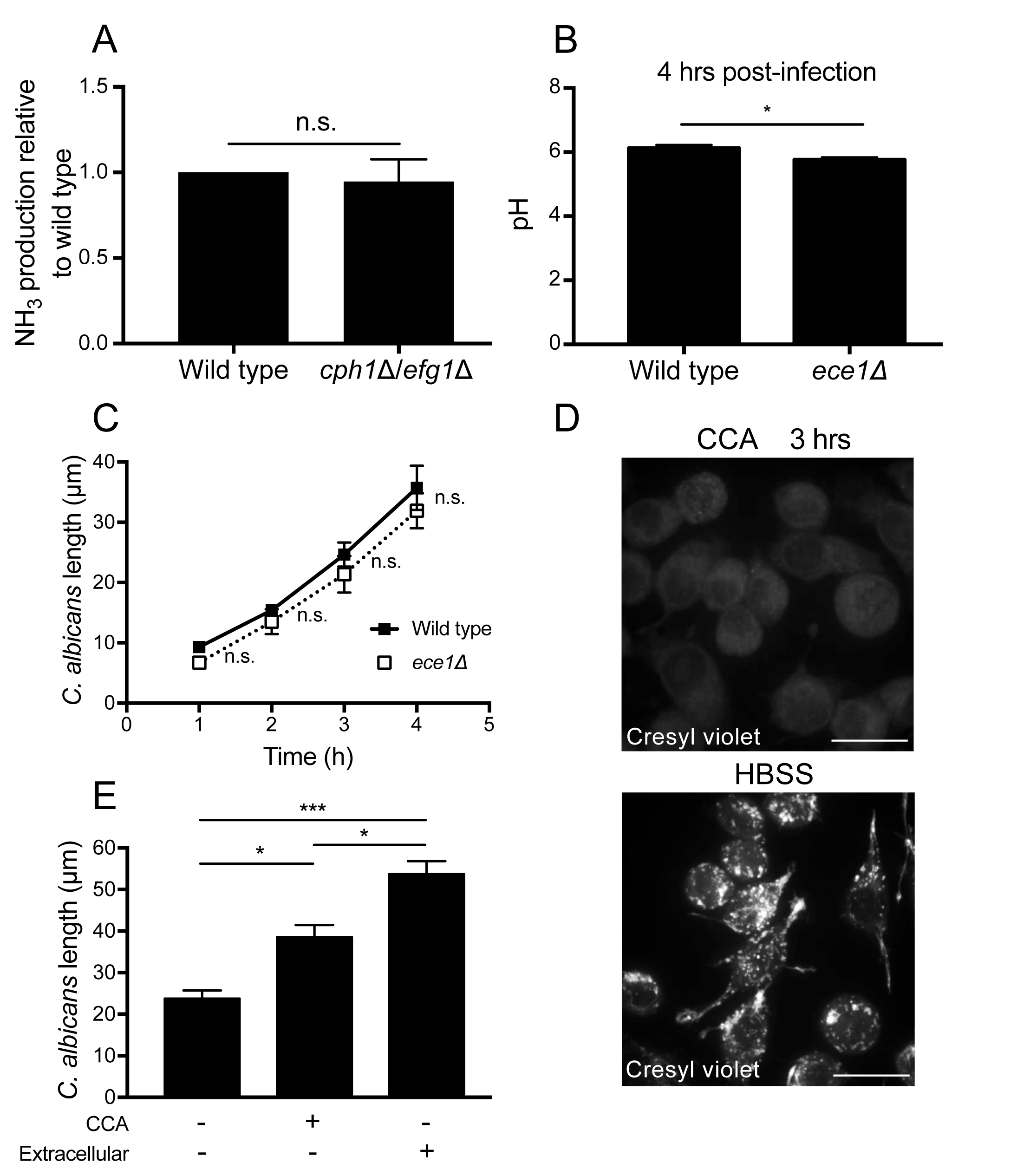

Supplement: FIG S2 [file mbo004184059sf2.tif]

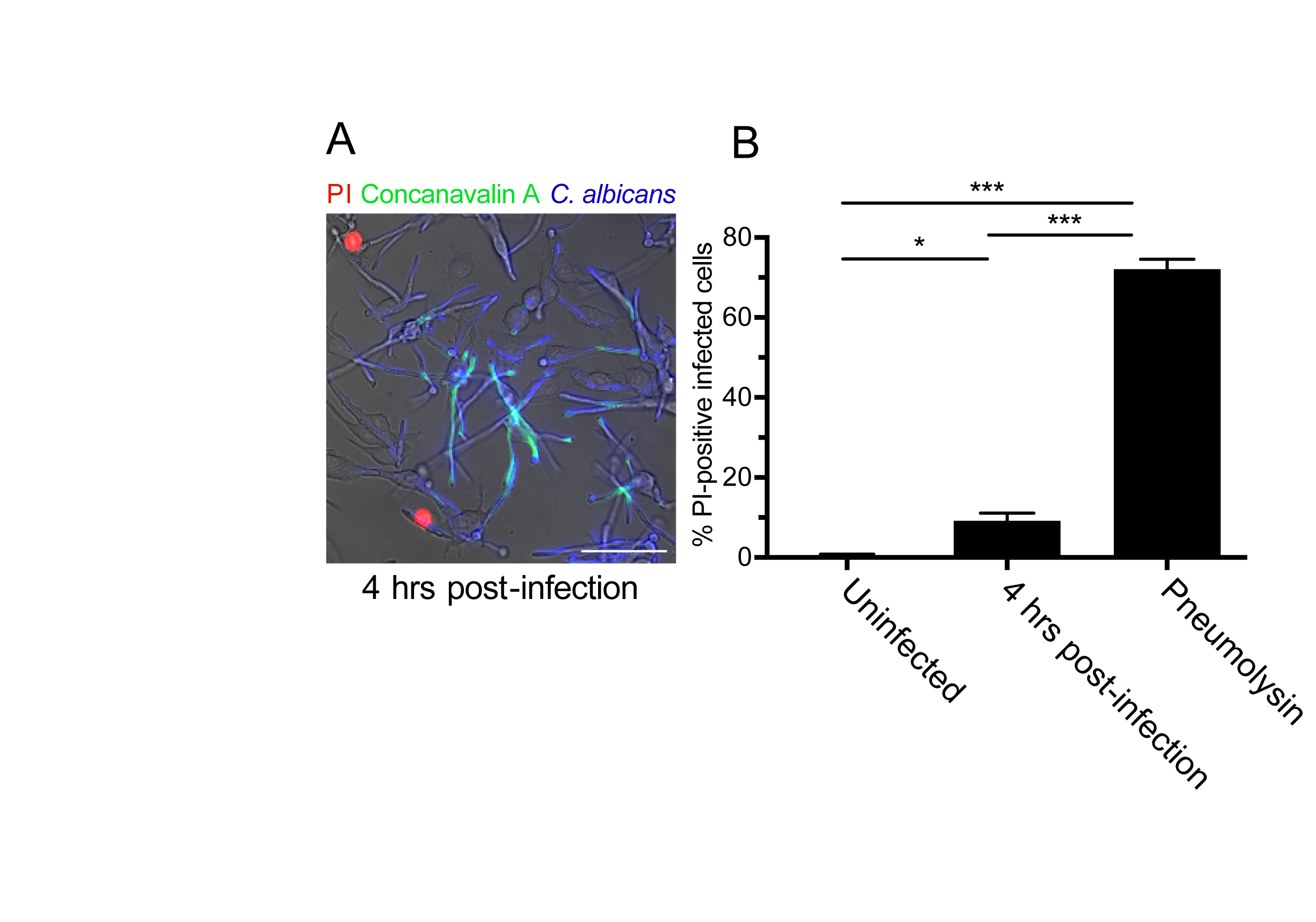

Supplement: FIG S3 [file mbo004184059sf3.tif]
