# Supplementary material for: Candida albicans Hyphal Expansion Causes Phagosomal Membrane Damage and Luminal Alkalinization
Source: mBio. 2018 Sep 11;9(5):e01226-18. doi: 10.1128/mBio.01226-18 (PMC6134096; doi:10.1128/mBio.01226-18)
Supplement: TABLE S1 [file mbo004184059st1.docx]

Table S1. PCR Primers used to generate and verify the Ece1 expressing yeast-locked strain (*cph1∆/efg1∆ + pENO1-ECE1*)**.**

| **Primer Name** | **Sequence (5’-3’)** |
| --- | --- |
| ACT1-F | tcagaccagctgatttaggtttg |
| ACT1-R | gtgaacaatggatggaccag |
| ECE1-F | atcgaaaatgccaagagag |
| ECE1-R | agcattttcaataccgacag |
| ECE1_ENO1_PR | aattctggagcatggtggatgatggcagcttgagaagataaagcaaaaacagtagcacaggcaattttggagaatttcattgttgtaatattcctgaattatc |
| ECE1_ENO1_PF | agcgaagtaagacatattacaaaaatttccagccactattttgtacctgtaactttcaaaaatgattacactttttgactcgttagtatcgaatcgacagc |
| ENO1p-S | ttgataattcaggaatattacaac |
| ECE1-IR | gtagaagcaacagaagtcatg |
