# Supplementary material for: Candida albicans Hyphal Expansion Causes Phagosomal Membrane Damage and Luminal Alkalinization
Source: mBio. 2018 Sep 11;9(5):e01226-18. doi: 10.1128/mBio.01226-18 (PMC6134096; doi:10.1128/mBio.01226-18)
Supplement: TABLE S2 [file mbo004184059st2.docx]

Table S2. *C. albicans* strains used in this study.

| **Strain name** | **Genotype** | **Strain description** | **Reference** | **Morphology** |
| --- | --- | --- | --- | --- |
| SC5314 | - | Clinical isolate (reference strain) | PMID:6394964 | Can form hyphae in hyphae-inducing conditions |
| Wild type  (BWP17/CIp30) | *ura3*::λimm434/*ura3*::λimm434  *iro1*::λimm434/*iro1*::λimm434  *his1*::*hisG*/*his1*::*hisG*  *arg4*::*hisG*/*arg4*::*hisG*  *RPS1*/*rps1*::(*URA3*-*HIS1*-*ARG4*) | Isogenic wild type derived from SC5314 | (Zakikhany *et al.*, 2007) | Can form hyphae in hyphae-inducing conditions |
| *ece1∆* | *ura3*::λimm434/*ura3*::λimm434  *iro1*::λimm434/*iro1*::λimm434  *his1*::*hisG*/*his1*::*hisG*  *arg4*::*hisG*/*arg4*::*hisG*  *ece1*::*HIS1*/*ece1*::*ARG4*  *RPS1*/*rps1*::*URA3* | Deletion mutant of *ECE1.*  *ECE1* encodes Candidalysin, peptide toxin | (Moyes *et al.*, 2016) | Can form hyphae in hyphae-inducing conditions |
| *cph1∆*/*efg1∆* | *cph1::hisG/cph1::hisG*  *efg1::hisG/efg1::hisG*  *RPS1/rps1::URA3* | Yeast locked mutant. Cph1 and Efg1 are transcription factors required for filamentation | (Wartenberg *et al.*, 2014) | Yeast-locked |
| *cph1∆/efg1∆ + pENO1-ECE1* | *cph1*::*hisG*/*cph1*::*hisG*  *efg1*::*hisG*/*efg1*::*hisG-URA3-hisG*  *ECE1*/*ece1*::*pENO1*-*ECE1* | Yeast-locked strain over-expressing *ECE1* under the control of the constitutive *ENO1* promoter | This study | Yeast-locked |
